# [1-(1*H*-Imidazo[4,5-*f*][1,10]phenanthro­lin-2-yl)naphthalen-2-ol-κ^2^ *N* ^7^,*N* ^8^]diiodidomercury(II)

**DOI:** 10.1107/S1600536811037160

**Published:** 2011-09-17

**Authors:** Tian-Le Li

**Affiliations:** a Physics Department, School of Science, Guangdong University of Petrochemical Technology, Maoming 525000, People’s Republic of China

## Abstract

In the title compound, [HgI_2_(C_23_H_14_N_4_O)], the Hg^II^ atom is four-coordinated by two N atoms from one 1-(1*H*-imidazo[4,5-*f*][1,10]phenanthrolin-2-yl)naphthalen-2-ol ligand and by two I atoms in a distorted tetra­hedral environment. An intra­molecular O—H⋯N hydrogen bond stabilizes the mol­ecular conformation and an inter­molecular N—H⋯I inter­action stabilizes the crystal packing.

## Related literature

For information about the organic ligand 1-(1*H*-imidazo[4,5-*f*][1,10]phenanthrolin-2-yl)naphthalen-2-ol), see: Wang *et al.* (2010 ▶).
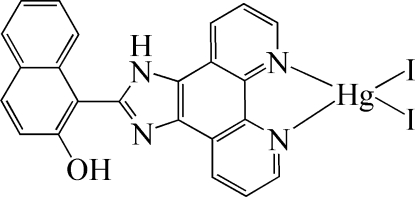


## Experimental

### 

#### Crystal data

[HgI_2_(C_23_H_14_N_4_O)]
*M* *_r_* = 816.77
Monoclinic, 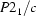

*a* = 14.4271 (10) Å
*b* = 7.3026 (5) Å
*c* = 21.1337 (15) Å
β = 94.472 (1)°
*V* = 2219.8 (3) Å^3^
*Z* = 4
Mo *K*α radiation
μ = 9.74 mm^−1^
*T* = 293 K
0.17 × 0.14 × 0.12 mm

#### Data collection

Bruker SMART APEX diffractometer
Absorption correction: multi-scan (*SADABS*; Sheldrick, 1996 ▶) *T*_min_ = 0.41, *T*_max_ = 0.64
11586 measured reflections
4351 independent reflections
3217 reflections with *I* > 2σ(*I*)
*R*_int_ = 0.031

#### Refinement

*R*[*F* ^2^ > 2σ(*F* ^2^)] = 0.029
*wR*(*F* ^2^) = 0.057
*S* = 1.01
4351 reflections
284 parameters
H atoms treated by a mixture of independent and constrained refinement
Δρ_max_ = 0.67 e Å^−3^
Δρ_min_ = −0.84 e Å^−3^

### 

Data collection: *SMART* (Bruker, 1997 ▶); cell refinement: *SAINT* (Bruker, 1997 ▶); data reduction: *SAINT*; program(s) used to solve structure: *SHELXS97* (Sheldrick, 2008 ▶); program(s) used to refine structure: *SHELXL97* (Sheldrick, 2008 ▶); molecular graphics: *XP* in *SHELXTL* (Sheldrick, 2008 ▶); software used to prepare material for publication: *SHELXL97*.

## Supplementary Material

Crystal structure: contains datablock(s) global, I. DOI: 10.1107/S1600536811037160/bt5643sup1.cif

Structure factors: contains datablock(s) I. DOI: 10.1107/S1600536811037160/bt5643Isup2.hkl

Additional supplementary materials:  crystallographic information; 3D view; checkCIF report

## Figures and Tables

**Table 1:** Hydrogen-bond geometry (Å, °)

*D*—H⋯*A*	*D*—H	H⋯*A*	*D*⋯*A*	*D*—H⋯*A*
N3—H3*A*⋯I1^i^	0.86	3.05	3.896 (4)	167
O1—H1*A*⋯N4	0.91 (8)	1.77 (8)	2.623 (6)	155 (7)

Symmetry code: (i) 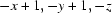.

**Fig. 1.**
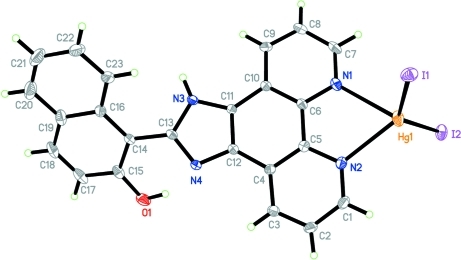
Perspective view of the title compound, showing the atomic numbering scheme. Displacement ellipsoids are drawn at the 30% probability level.

## supplementary crystallographic information

### Comment

The ligand 1-(1H-imidazo[4,5-f][1,10]phenanthrolin-2-yl)naphthalen-2-ol) is a  N-donor ligand and has excellent coordinating ability (Wang *et al.*, 2010). In this work, we selected it as an N-donor chelating ligand, generating a new Hg^II^ complex.

In the compound, the central Hg^II^ atom is four-coordinated by two N atoms from one organic ligand, and two I atoms in a distorted tetrahedral sphere. N-H···I and O-H···N H-bonding interactions   stabilize the crystal structure and the molecular conformation.

### Experimental

A mixture of HgI_2_ (0.5 mmol) and 1-(1H-imidazo[4,5-f][1,10]phenanthrolin-2-yl)naphthalen-2-ol) (0.5 mmol) in 8 mL distilled water was heated at 462 K in a Teflon-lined stainless steel autoclave for seven days. The reaction system was then slowly cooled to room temperature. Pale yellow crystals of the title compound suitable for single crystal X-ray diffraction analysis were collected from the final reaction system by filtration, washed several times with distilled water and dried in air at ambient temperature. Yield: 15% based on Hg(II).

### Refinement

H atoms  bonded to N and C were positioned geometrically (C-H = 0.93 Å) and refined as riding, with Uiso(H)=1.2U_eq_(carrier). The H atom bonded to O was freely refined.

### Crystal data

**Table:**

[HgI_2_(C_23_H_14_N_4_O)]	*F*(000) = 1496
*M**_r_* = 816.77	*D*_x_ = 2.444 Mg m^−^^3^
Monoclinic, *P*2_1_/*c*	Mo *K*α radiation, λ = 0.71073 Å
Hall symbol: -P 2ybc	Cell parameters from 4351 reflections
*a* = 14.4271 (10) Å	θ = 1.9–26.0°
*b* = 7.3026 (5) Å	µ = 9.74 mm^−^^1^
*c* = 21.1337 (15) Å	*T* = 293 K
β = 94.472 (1)°	Block, pale yellow
*V* = 2219.8 (3) Å^3^	0.17 × 0.14 × 0.12 mm
*Z* = 4	

### Data collection

**Table:**

Bruker SMART APEX diffractometer	4351 independent reflections
Radiation source: fine-focus sealed tube	3217 reflections with *I* > 2σ(*I*)
graphite	*R*_int_ = 0.031
φ and ω scans	θ_max_ = 26.0°, θ_min_ = 1.9°
Absorption correction: multi-scan (*SADABS*; Sheldrick, 1996)	*h* = −16→17
*T*_min_ = 0.41, *T*_max_ = 0.64	*k* = −7→9
11586 measured reflections	*l* = −25→26

### Refinement

**Table:**

Refinement on *F*^2^	Primary atom site location: structure-invariant direct methods
Least-squares matrix: full	Secondary atom site location: difference Fourier map
*R*[*F*^2^ > 2σ(*F*^2^)] = 0.029	Hydrogen site location: inferred from neighbouring sites
*wR*(*F*^2^) = 0.057	H atoms treated by a mixture of independent and constrained refinement
*S* = 1.01	*w* = 1/[σ^2^(*F*_o_^2^) + (0.0207*P*)^2^ + 0.5359*P*] where *P* = (*F*_o_^2^ + 2*F*_c_^2^)/3
4351 reflections	(Δ/σ)_max_ = 0.002
284 parameters	Δρ_max_ = 0.67 e Å^−^^3^
0 restraints	Δρ_min_ = −0.84 e Å^−^^3^

### Special details

**Table:**

Geometry. All esds (except the esd in the dihedral angle between two l.s. planes) are estimated using the full covariance matrix. The cell esds are taken into account individually in the estimation of esds in distances, angles and torsion angles; correlations between esds in cell parameters are only used when they are defined by crystal symmetry. An approximate (isotropic) treatment of cell esds is used for estimating esds involving l.s. planes.
Refinement. Refinement of F^2^ against ALL reflections. The weighted R-factor wR and goodness of fit S are based on F^2^, conventional R-factors R are based on F, with F set to zero for negative F^2^. The threshold expression of F^2^ > 2sigma(F^2^) is used only for calculating R-factors(gt) etc. and is not relevant to the choice of reflections for refinement. R-factors based on F^2^ are statistically about twice as large as those based on F, and R- factors based on ALL data will be even larger.

### Fractional atomic coordinates and isotropic or equivalent isotropic displacement parameters (Å^2^)

**Table:**

	*x*	*y*	*z*	*U*_iso_*/*U*_eq_	
C2	0.8132 (4)	0.3909 (8)	−0.2038 (3)	0.0445 (14)	
H2	0.8549	0.3558	−0.2330	0.053*	
C7	0.6301 (4)	0.7203 (7)	0.0288 (2)	0.0479 (15)	
H7	0.6592	0.7552	0.0677	0.058*	
C8	0.5367 (4)	0.7611 (8)	0.0162 (2)	0.0465 (14)	
H8	0.5047	0.8245	0.0459	0.056*	
C9	0.4920 (4)	0.7077 (7)	−0.0400 (2)	0.0387 (13)	
H9	0.4290	0.7309	−0.0488	0.046*	
C10	0.5440 (3)	0.6156 (6)	−0.0847 (2)	0.0292 (11)	
C11	0.5070 (3)	0.5568 (6)	−0.1458 (2)	0.0291 (11)	
C13	0.4226 (3)	0.4818 (6)	−0.2345 (2)	0.0308 (11)	
C14	0.3454 (3)	0.4641 (7)	−0.2832 (2)	0.0344 (12)	
C15	0.3641 (4)	0.5059 (7)	−0.3451 (2)	0.0434 (13)	
C16	0.2540 (4)	0.4097 (8)	−0.2700 (3)	0.0424 (14)	
C17	0.2914 (5)	0.5155 (8)	−0.3934 (3)	0.0595 (18)	
H17	0.3039	0.5523	−0.4340	0.071*	
C18	0.2032 (5)	0.4714 (8)	−0.3811 (3)	0.0593 (18)	
H18	0.1557	0.4787	−0.4133	0.071*	
C19	0.1830 (4)	0.4155 (8)	−0.3208 (3)	0.0546 (17)	
C20	0.0924 (5)	0.3593 (10)	−0.3092 (4)	0.083 (2)	
H20	0.0451	0.3663	−0.3416	0.099*	
C21	0.0735 (5)	0.2951 (11)	−0.2514 (5)	0.090 (3)	
H21	0.0131	0.2589	−0.2448	0.108*	
C22	0.1429 (5)	0.2820 (10)	−0.2013 (3)	0.075 (2)	
H22	0.1294	0.2351	−0.1621	0.091*	
C23	0.2314 (4)	0.3399 (8)	−0.2110 (3)	0.0563 (17)	
H23	0.2775	0.3327	−0.1778	0.068*	
N3	0.4179 (3)	0.5490 (5)	−0.17388 (18)	0.0316 (10)	
H3A	0.3681	0.5804	−0.1567	0.038*	
O1	0.4506 (3)	0.5473 (6)	−0.36141 (19)	0.0563 (11)	
H1A	0.488 (5)	0.518 (10)	−0.326 (4)	0.12 (3)*	
C1	0.8439 (4)	0.4232 (8)	−0.1411 (3)	0.0466 (14)	
H1	0.9066	0.4048	−0.1290	0.056*	
C3	0.7213 (4)	0.4112 (7)	−0.2222 (2)	0.0395 (13)	
H3	0.6990	0.3871	−0.2639	0.047*	
C4	0.6609 (3)	0.4691 (6)	−0.1774 (2)	0.0313 (11)	
C5	0.6970 (3)	0.5071 (6)	−0.1156 (2)	0.0323 (11)	
C6	0.6386 (3)	0.5855 (6)	−0.0694 (2)	0.0298 (11)	
C12	0.5631 (3)	0.4922 (6)	−0.1909 (2)	0.0301 (11)	
N1	0.6800 (3)	0.6338 (6)	−0.01211 (19)	0.0390 (11)	
N2	0.7895 (3)	0.4787 (5)	−0.09743 (19)	0.0375 (10)	
N4	0.5108 (3)	0.4486 (5)	−0.24611 (18)	0.0316 (10)	
Hg1	0.837535 (16)	0.50252 (3)	0.013119 (11)	0.05187 (8)	
I1	0.78377 (3)	0.23045 (5)	0.087966 (19)	0.05635 (12)	
I2	0.96077 (3)	0.77042 (5)	0.026201 (19)	0.05224 (12)	

### Atomic displacement parameters (Å^2^)

**Table:**

	*U*^11^	*U*^22^	*U*^33^	*U*^12^	*U*^13^	*U*^23^
C2	0.039 (3)	0.053 (4)	0.043 (3)	0.006 (3)	0.014 (3)	0.005 (3)
C7	0.070 (4)	0.047 (4)	0.026 (3)	−0.011 (3)	0.003 (3)	−0.006 (3)
C8	0.064 (4)	0.047 (4)	0.029 (3)	0.001 (3)	0.010 (3)	−0.004 (3)
C9	0.041 (3)	0.041 (3)	0.035 (3)	0.005 (2)	0.013 (2)	0.003 (3)
C10	0.035 (3)	0.026 (3)	0.027 (3)	−0.002 (2)	0.003 (2)	0.004 (2)
C11	0.029 (3)	0.033 (3)	0.025 (3)	0.000 (2)	0.003 (2)	0.001 (2)
C13	0.042 (3)	0.026 (3)	0.025 (2)	−0.003 (2)	0.005 (2)	0.002 (2)
C14	0.040 (3)	0.037 (3)	0.026 (3)	0.000 (2)	0.002 (2)	−0.003 (2)
C15	0.060 (4)	0.033 (3)	0.035 (3)	0.006 (3)	−0.007 (3)	−0.003 (3)
C16	0.037 (3)	0.044 (3)	0.046 (3)	0.007 (3)	0.000 (3)	−0.015 (3)
C17	0.099 (6)	0.042 (4)	0.034 (3)	0.013 (4)	−0.015 (3)	−0.005 (3)
C18	0.073 (5)	0.049 (4)	0.050 (4)	0.018 (3)	−0.029 (3)	−0.016 (3)
C19	0.046 (4)	0.044 (4)	0.070 (5)	0.015 (3)	−0.020 (3)	−0.021 (3)
C20	0.043 (5)	0.086 (6)	0.115 (7)	0.014 (4)	−0.016 (4)	−0.039 (5)
C21	0.037 (4)	0.095 (6)	0.140 (8)	−0.002 (4)	0.018 (5)	−0.048 (6)
C22	0.060 (5)	0.094 (6)	0.077 (5)	−0.018 (4)	0.033 (4)	−0.033 (4)
C23	0.049 (4)	0.071 (4)	0.051 (4)	−0.009 (3)	0.013 (3)	−0.024 (3)
N3	0.035 (2)	0.034 (2)	0.026 (2)	−0.0019 (18)	0.0057 (18)	−0.0040 (18)
O1	0.071 (3)	0.066 (3)	0.033 (2)	−0.006 (2)	0.012 (2)	0.001 (2)
C1	0.029 (3)	0.058 (4)	0.052 (4)	0.003 (3)	0.004 (3)	0.007 (3)
C3	0.042 (3)	0.042 (3)	0.035 (3)	0.004 (2)	0.005 (3)	−0.001 (3)
C4	0.033 (3)	0.030 (3)	0.031 (3)	0.000 (2)	0.006 (2)	0.002 (2)
C5	0.034 (3)	0.024 (3)	0.038 (3)	−0.002 (2)	0.002 (2)	0.003 (2)
C6	0.039 (3)	0.027 (3)	0.024 (3)	−0.006 (2)	0.002 (2)	0.006 (2)
C12	0.034 (3)	0.028 (3)	0.028 (3)	−0.002 (2)	0.002 (2)	0.001 (2)
N1	0.047 (3)	0.038 (3)	0.031 (2)	−0.006 (2)	−0.004 (2)	0.003 (2)
N2	0.032 (2)	0.040 (3)	0.040 (2)	0.000 (2)	−0.0001 (19)	0.004 (2)
N4	0.033 (2)	0.037 (2)	0.025 (2)	−0.0007 (18)	0.0039 (18)	−0.0021 (18)
Hg1	0.04968 (14)	0.05769 (16)	0.04649 (14)	−0.01629 (12)	−0.00742 (10)	0.00740 (13)
I1	0.0555 (2)	0.0551 (3)	0.0607 (3)	0.00396 (19)	0.0187 (2)	0.0143 (2)
I2	0.0464 (2)	0.0458 (2)	0.0623 (3)	−0.00957 (18)	−0.00981 (18)	0.0071 (2)

### Geometric parameters (Å, °)

**Table:**

C2—C3	1.361 (7)	C18—H18	0.9300
C2—C1	1.385 (7)	C19—C20	1.410 (9)
C2—H2	0.9300	C20—C21	1.357 (11)
C7—N1	1.327 (6)	C20—H20	0.9300
C7—C8	1.385 (8)	C21—C22	1.403 (10)
C7—H7	0.9300	C21—H21	0.9300
C8—C9	1.363 (7)	C22—C23	1.375 (8)
C8—H8	0.9300	C22—H22	0.9300
C9—C10	1.420 (6)	C23—H23	0.9300
C9—H9	0.9300	N3—H3A	0.8600
C10—C6	1.396 (6)	O1—H1A	0.91 (8)
C10—C11	1.425 (6)	C1—N2	1.322 (6)
C11—N3	1.375 (6)	C1—H1	0.9300
C11—C12	1.381 (6)	C3—C4	1.402 (6)
C13—N4	1.337 (6)	C3—H3	0.9300
C13—N3	1.378 (6)	C4—C5	1.396 (7)
C13—C14	1.463 (6)	C4—C12	1.427 (6)
C14—C15	1.391 (7)	C5—N2	1.374 (6)
C14—C16	1.425 (7)	C5—C6	1.456 (6)
C15—O1	1.355 (7)	C6—N1	1.354 (6)
C15—C17	1.407 (8)	C12—N4	1.376 (6)
C16—C23	1.409 (8)	N1—Hg1	2.486 (4)
C16—C19	1.425 (7)	N2—Hg1	2.391 (4)
C17—C18	1.358 (9)	Hg1—I2	2.6435 (4)
C17—H17	0.9300	Hg1—I1	2.6912 (5)
C18—C19	1.389 (9)		
			
C3—C2—C1	119.2 (5)	C20—C21—H21	119.3
C3—C2—H2	120.4	C22—C21—H21	119.3
C1—C2—H2	120.4	C23—C22—C21	118.9 (7)
N1—C7—C8	123.2 (5)	C23—C22—H22	120.6
N1—C7—H7	118.4	C21—C22—H22	120.6
C8—C7—H7	118.4	C22—C23—C16	121.7 (6)
C9—C8—C7	119.4 (5)	C22—C23—H23	119.2
C9—C8—H8	120.3	C16—C23—H23	119.2
C7—C8—H8	120.3	C11—N3—C13	107.6 (4)
C8—C9—C10	118.5 (5)	C11—N3—H3A	126.2
C8—C9—H9	120.8	C13—N3—H3A	126.2
C10—C9—H9	120.8	C15—O1—H1A	103 (5)
C6—C10—C9	118.6 (5)	N2—C1—C2	123.9 (5)
C6—C10—C11	116.9 (4)	N2—C1—H1	118.1
C9—C10—C11	124.5 (4)	C2—C1—H1	118.1
N3—C11—C12	105.5 (4)	C2—C3—C4	118.9 (5)
N3—C11—C10	132.4 (4)	C2—C3—H3	120.5
C12—C11—C10	122.1 (4)	C4—C3—H3	120.5
N4—C13—N3	110.6 (4)	C5—C4—C3	119.0 (4)
N4—C13—C14	122.6 (4)	C5—C4—C12	117.1 (4)
N3—C13—C14	126.5 (4)	C3—C4—C12	123.9 (5)
C15—C14—C16	119.7 (5)	N2—C5—C4	121.1 (4)
C15—C14—C13	116.7 (4)	N2—C5—C6	118.1 (4)
C16—C14—C13	123.6 (4)	C4—C5—C6	120.8 (4)
O1—C15—C14	122.6 (5)	N1—C6—C10	121.4 (5)
O1—C15—C17	117.0 (5)	N1—C6—C5	117.7 (4)
C14—C15—C17	120.3 (6)	C10—C6—C5	120.9 (4)
C23—C16—C19	118.4 (5)	N4—C12—C11	110.6 (4)
C23—C16—C14	123.7 (5)	N4—C12—C4	127.6 (4)
C19—C16—C14	117.8 (5)	C11—C12—C4	121.7 (4)
C18—C17—C15	120.4 (6)	C7—N1—C6	118.8 (5)
C18—C17—H17	119.8	C7—N1—Hg1	125.3 (3)
C15—C17—H17	119.8	C6—N1—Hg1	115.0 (3)
C17—C18—C19	120.8 (6)	C1—N2—C5	117.9 (4)
C17—C18—H18	119.6	C1—N2—Hg1	124.2 (3)
C19—C18—H18	119.6	C5—N2—Hg1	117.6 (3)
C18—C19—C20	120.6 (6)	C13—N4—C12	105.6 (4)
C18—C19—C16	120.6 (6)	N2—Hg1—N1	68.14 (14)
C20—C19—C16	118.8 (7)	N2—Hg1—I2	107.31 (9)
C21—C20—C19	120.8 (7)	N1—Hg1—I2	109.47 (10)
C21—C20—H20	119.6	N2—Hg1—I1	116.46 (9)
C19—C20—H20	119.6	N1—Hg1—I1	96.33 (9)
C20—C21—C22	121.4 (7)	I2—Hg1—I1	135.010 (15)

### Hydrogen-bond geometry (Å, °)

**Table:**

*D*—H···*A*	*D*—H	H···*A*	*D*···*A*	*D*—H···*A*
N3—H3A···I1^i^	0.86	3.05	3.896 (4)	167.
O1—H1A···N4	0.91 (8)	1.77 (8)	2.623 (6)	155 (7)

Symmetry codes: (i) −*x*+1, −*y*+1, −*z*.
